# Atypical periosteal osteoid osteoma: a case report

**DOI:** 10.1186/1757-1626-2-124

**Published:** 2009-02-04

**Authors:** SS Suresh, V Rani

**Affiliations:** 1 Department of Orthopaedics, Ibri Regional Referral Hospital, PO Box 46, Ibri 516, Sultanate of Oman; 2 Department of Anaesthesia, Ibri Regional Referral Hospital, PO Box 46, Ibri 516, Sultanate of Oman

## Abstract

Osteoid osteoma is a benign osteoblastic tumor usually seen in adolescent and young males. In the paediatric age group, since the history may be difficult to elicit, there are often problems in early diagnosis. The author reports an unusual presentation of osteoid osteoma in a ten-year-old girl, which could not be diagnosed by conventional X-rays and CT scan.

## Background

Osteoid osteoma first described by Jaffe 1n 1935, is a benign osteoblastic tumour, mostly seen in adolescent and young males[1]. The lesions being more common in the lower extremity, children present with painful limping[1]. Conventional radiographs are effective in diagnosis, the radiological picture is of a central radiolucent area (nidus) surrounded by an area of cortical thickening. The imaging modality of choice is CT scan which is considered far superior to an MRI in diagnosis[1,2]. Tumors present in its classic form only in two thirds of the patients[3]. Subperiosteal location of the lesion can make difficulties in diagnosis. There are reports that osteoid osteomas arose in the subperiosteal region[4] initially and later on become cortical or intramedullary.

## Case report

A ten-year-old girl was seen in the orthopedic clinic with recurrent episodes of pain left ankle of around five months duration. She was not having diurnal variation of her symptoms and her medical history was unremarkable. There was a small tender swelling over the posteromedial aspect of the left ankle between the postero medial border of tibia and the tibialis posterior tendon. Movements of the ankle joint were free. Her blood parameters were normal.

**Figure 1:**
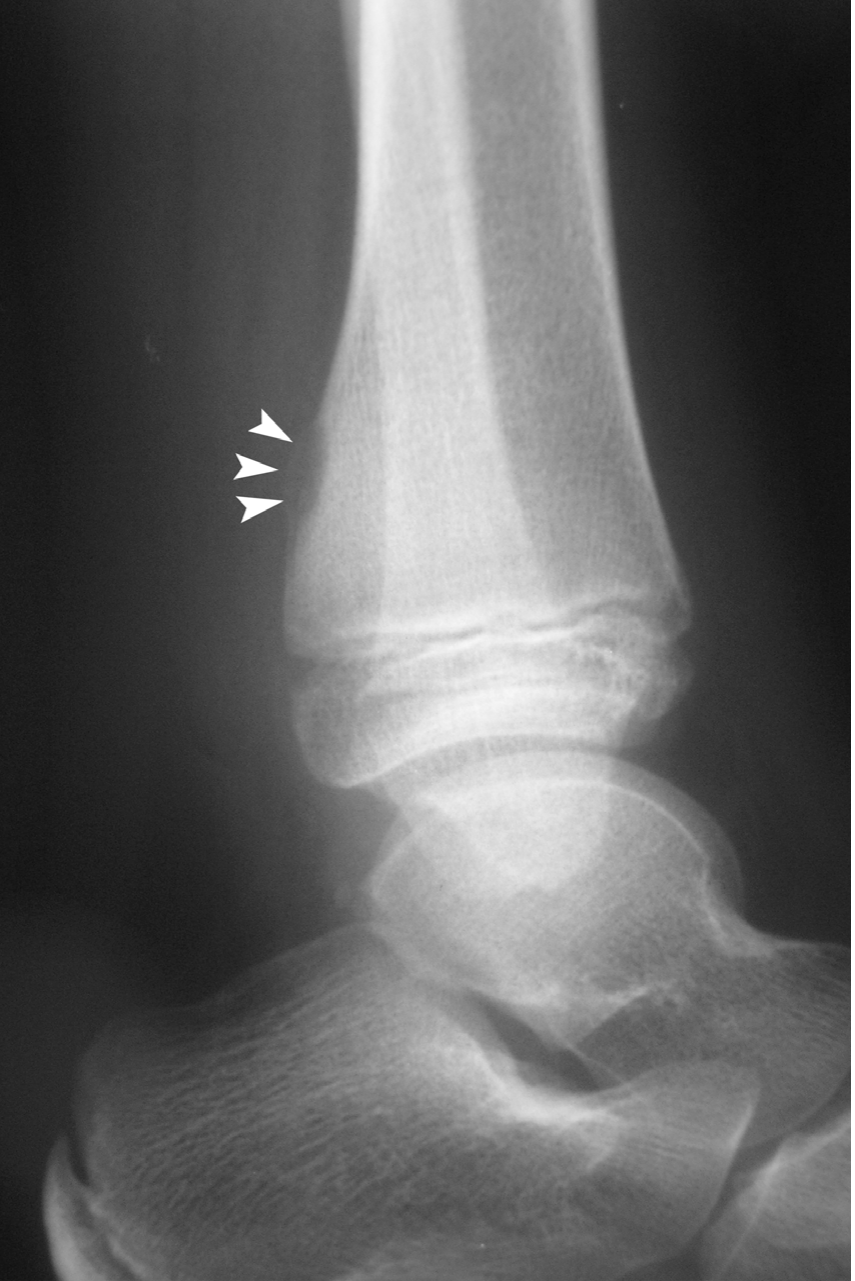
**X-ray of the left ankle showing periosteal lesion with thin outer shell (white arrow heads)**.

Routine X-ray of the left ankle showed a ballooned out lesion over the posterior aspect of the left tibia close to the growth plate, with a thin outer shell. There was no sclerosis around the lesion and no calcification could be seen inside. Ultrasound scan of the lesion didn't reveal any gross abnormality (see Figure 1).

**Figure 2:**
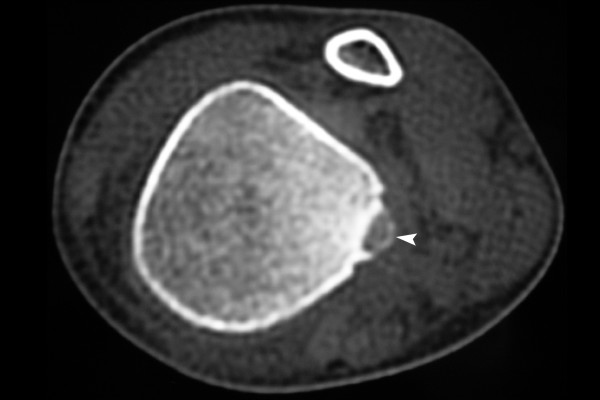
**CT scan showing the same lesion as in the X-ray, with doubtful calcification inside(white arrow head)**.

A CT scan was done which showed an elliptical hypodense area in the posterior distal tibia with only minimal sclerosis. There was no soft tissue component and periosteal reaction. The radiological diagnosis was fibrous cortical defect (see Figure 2).

The patient was advised surgery as she was symptomatic. Through a postero medial incision the lesion was approached after retracting the tibialis posterior tendon and the neuro-vascular bundle. Ballooning of the distal posterior tibia was found, which was curetted. Punctuate bleeding tissue was removed with high speed burr. Histopathology report was consistent with osteoid osteoma. The patient was asymptomatic at one year follow up.

## Discussion

Clinical symptoms and radiological findings are enough to make the diagnosis of osteoid osteoma at presentation. The location of the osteoid osteoma may be intra cortical, subperiosteal, endosteal, or medullary. The lesion is most commonly located in the cortex of the long bones with dense reactive sclerosis. Presence of area of sclerosis around the lucent area, due to reactive bone formation is typical of osteoid osteoma[2]. The nidus on maturity may be radio dense due to mineralization. The nidus is better demonstrated in a CT scan than in an MRI[1,2]. CT scanning with 2 mm cuts delineates the nidus properly. CT scan shows a nidus which is a well-defined area of low attenuation which is surrounded by an area of high attenuation of reactive sclerosis[1].

Radiographic appearance may vary with location of the tumor[1]. The tumor presents in its typical form only in two thirds of the patients[3] as the intial radiographs may be misleading. The difficulties in diagnose arose from the absence of reactive new bone formation in atypical sites, as in our case.

Radiographs in nondiaphyseal osteoid osteomas may be misleading and the tumor may be missed in most of the cases. In a small series by Davidson et al there was a delay of six months in making the diagnosis because of the atypical radiographic appearance[3]. The delay in diagnosis can range from six months to two years. Most of the osteoid osteomas are cortical, which is associated with more marked reactive formation compared to medullary and subperiosteal osteoid osteomas. Nidus in cancellous bone may be difficult to visualize as there is less periosteal reaction and new bone formation. Subperiosteal lesion shows less periosteal bone formation than the cortical lesion[5]. Authors found that subperiosteal osteoid osteoma like any tumors may erode into the cortex beneath[5]. Subperiosteal osteoid osteoma as reported by Shankman et al is a rare tumor on the surface of the bone. There is scanty mineralization and less reactive sclerosis of the adjacent bone.

Contrary to this Kayser[1] believes that most osteoid osteomas arise in the subperiosteal location and later appear intra cortical or intramedullary. The authors propose two mechanisms for this transition, continuous remodeling of the bone causing shift of the nidus, and differential remodeling and cortical drift of immature bone. The frequency of osteoid osteoma arising in the subperiosteal location is not that rare.

Osteoid osteoma tends to regress over a period of time even without treatment. Salicylates can accelerate healing in osteoid osteoma and is a good diagnostic and therapeutic consideration[1]. The standard treatment of osteoid osteoma is enbloc resection, though other modalities of treatment including radio ablation have been practiced. Patient operated with high speed burr has less morbidity compared with enbloc resection[1].

Subperiosteal osteoid osteomas produce atypical radiographic picture and the radiological features may be misleading. Subperiosteal osteoid osteoma is a rare lesion with atypical radiographic features. Our case was also not diagnosed as osteoid osteoma as the location of the tumor was atypical, and there was no reactive new bone formation.

## Consent

Written informed consent was obtained from the patient for publication of this case report and accompanying images. A copy of the written consent is available for review by the Editor-in-Chief of this journal

## Competing interests

The authors declare that they have no competing interests.

## Authors' contributions

SSS was responsible for diagnosis and management of the case, analysis of data, preparation of manuscript .VR was responsible for management of the case, analysis of data and preparation of manuscript.
